# How Youth of Color Create Communities of Hope: Connecting Advocacy, Activity, and Neighborhood Change

**DOI:** 10.3390/ijerph18063133

**Published:** 2021-03-18

**Authors:** Anna J. Kim, Jasmine Jones-Bynes, Nisha Botchwey, Terry L. Conway

**Affiliations:** 1 Department of City Planning, San Diego State University, San Diego, CA 92182, USA; 2 City and Regional Planning, Georgia Institute of Technology, Atlanta, GA 30332, USA; jjonesbynes3@gatech.edu (J.J.-B.); nisha.botchwey@gatech.edu (N.B.); 3 Department of Family and Preventative Medicine, UC San Diego, San Diego, CA 92182, USA; tlconway@ucsd.edu

**Keywords:** youth participation, community engagement, neighborhood inequality, physical activity, advocacy, after-school programs

## Abstract

The primary aim of this paper was to assess the association of after-school club characteristics with changes in physical activity, nutrition, and attitudes in students of color after participating in the “YEAH!” Advocacy-based Physical Activity Program. We examine the strengths of school-based vs. non-school based programs in promoting feelings of self-efficacy and empowerment among students learning to become more physically active—and importantly, also test the strength of how programs that are more connected (to community-based partners) may contribute to students’ optimism around policy and public health as it directly affects them. This study examined differences in the youth advocacy training impact across four after-school club types: school-based with community partnerships, school-based without partnerships, non-school-based with community partners, and non-school-based clubs without partnerships. We measured improvements in youth’s “optimism for change”, “assertiveness” and “decision-making” as related to after school activities and found that non-school-based programs with community partners showed highest positive impact.

## 1. Introduction

A healthy environment is not an evenly accessible environment. Promoting youth advocacy in health interventions has positive implications for increasing physical activity behavior in youth and environmental change in communities [1,2]. However, no communities are created equal, to begin with, and this is one of the greatest challenges to overcoming physical barriers to activity and healthier neighborhoods. Communities vary in their accessibility to parks, public spaces, open spaces, and natural environmental amenities (beaches, mountains, deserts for example). Most low-income communities of color across the U.S. experience disproportionately lower access to all of the above. Pandemics and civil unrest have only exacerbated these existing inequalities of access, and quality, of physical space—particularly in urban areas [3]. Additionally, as we have seen recently, in times of crisis, this inequity of access is compounded by the social and physical effects of historic inequality in terms of health, leading to further harm and increased morbidity in African American and Latinx neighborhoods, specifically [4].

Our study of the efficacy of the Youth Engagement and Action for Health! (YEAH!) curriculum in low-income middle school students of color examined the impact of youth advocacy training for physical activity, environment, and policy (PSE) changes on intervention processes, youth psychosocial factors, participation, hypothesized drives of change, and proximal outcomes in youth participants [5]. We collaborated with 18 youth-serving organizations located across the United States. Specifically, we targeted recruitment of minority middle school youth through after-school programs, schools, community groups, and a church youth group located in low-income urban, suburban and rural communities. As planners and public health professionals, we were interested in how students’ engaging in the YEAH! curriculum, but also advocating for local or community-level policy changes around the health of their built environment, school, or neighborhoods, would relate to changes in their feelings of hope, empowerment, and belief in change. 

Taking the perspective of a young person, imagine that you live in a city and you see that your neighborhood parks, your playgrounds, and your sidewalks are not maintained. The few parks and public spaces you do make your way to when you are allowed to go to them, appear to be deteriorating before your eyes. You might ask yourself, is something wrong with my neighborhood? Why isn’t my neighborhood being shown care? Is something wrong with me? Parks without playgrounds, running tracks without pavement, basketball courts without hoops, were some of the many observations our students made about what was missing in their built environment and from what they saw as an important component of a “healthy community” in their neighborhood.

While other studies have linked historic race and class-based segregation as being connected to lower access to parks and public spaces [6,7] few have examined how hopeful, or not hopeful, young residents feel about future change in their environment and their larger communities. It is this quality of change, or optimism for change, that we examined in students who participated in an advocacy-based curriculum around improving health outcomes, located at middle schools and with after school-based programs around the country. The students we surveyed were from neighborhoods where many youth experience lower access to important spaces for safe and appropriate physical activity, and concerningly, lower levels of hope, or what we call “optimism for change”.

After-school programs (ASPs) grew in popularity and funding during the mid-1970s as a result of increased crime in cities and increased drug/alcohol use among teens. The general idea among educators, community leaders, and working parents was that neighborhoods were no longer safe for children to roam unsupervised while both parents were at work [8,9]. Initially, ASPs were provided by small community organizations such as religious institutions and community centers such as the YMCA. States began allocation school districts funding to provide school-based after-school programs. In 1994 the United States federal government approved the 21st Century Community Learning Centers program which provides funds to schools and community organizations that provide after-school and summer programs for youth. Funding to support ASP has increased tremendously, rising from 40 million in 1998 to 1.2 billion in 2019. Many challenges impact the effectiveness and reach of after-school programs including transportation, retention, and funding.

The importance of after-school programs is long studied. Researchers identify improved academic achievement [10,11], self-perceptions, social behaviors [9], and reduced drug use [12] as demonstrated benefits of after-school programs. A review of the benefits of after-school programs on Latino youth found that students who participated for at least 20 h per week had higher self-worth, greater ethnic identity, and fewer problems concentrating than students who did not [13]. A review of nearly seventy after-school programs found that the programs significantly increased students’ positive feelings and attitudes about themselves and their school [12]. A review of 1000 elementary schools and 4300 middle school students participating in school-based after-school programs found modest differences in social development between ASP students and non-ASP students [14]. 

One of the most telling benefits of funding for after-school programs is the decline in unsupervised youth. A nation-wide survey of after-school programs found that between 2004 and 2014 the number of children participating in after-school programming increased by 3 million. The United States average attendance for after-school programs is 18%, Idaho and Utah had the lowest attendance (less than 10%), while Washington, D.C. had the highest attendance (35%). Attendance is highest among elementary and middle school students and lowest among high-school students. In terms of program offerings, the majority of ASPs are sponsored by schools (54%), followed by national community organizations (18% Boys and Girls Clubs and 15% YMCA), followed by religious organizations (10%).

In addition to educational and social development, after-school programs have the potential to increase physical activity among participating youth. A national review of after-school program standards found that just 14 contained guidelines for the inclusion of physical activity, of those just 5 outlined standards, and 3 included physical activity as a core competency [15]. The standards included thirty- to sixty-minute physical activity requirements and limits of 60 min of screen-time. Absent of a national standard or policy for the inclusion of physical activity in after-school programs researchers concluded that mandatory physical education courses and short, frequent 10-min physical activity breaks during the school day present the maximum increase in physical activity among youth [16].

After-school programs have greater efficacy in nutrition among youth. Researchers found that after participating in a 12-week after-school program youth and parent’s consumption of fruit increased while consumption of fried foods decreased [17]. The existing literature indicates that school-based activities can improve physical activity and in a previous paper [18] we also found that a majority of students of color from varying backgrounds (African American, Latinx, Asian American, and Pacific Islander) and rural to urban settings improved their number of physically active days per week after participation in the YEAH! curriculum. 

## 2. Materials and Methods

Our study focuses on youth of color primarily from the above three racial groups across urban, suburban, and rural geographies to measure optimism among youth for future change, before and after participating in the program. We examine how what we learned from young peoples’ relationships to physical place and physical activity connects with optimism for change and assertiveness in advocating for themselves. We look closely at change in communities where students live and/or go to school in majority-minority places.

This paper seeks to explain whether two general characteristics of clubs—the type of club (whether that club is school-based or non-school based) and the presence of a community partnership (yes or no) are associated with greater changes in YEAH! constructs after participating in the advocacy intervention. In this paper, we assess the influence of the above after school program characteristics on the magnitude of impacts on positive or negative change in student physical activity, nutrition, and self-efficacy related attitudes and behaviors after participating in the YEAH! Program. The study examines these differences in terms of trend analysis across four youth club types: school-based with community partnerships such as non-governmental organizations, school-based without community partners, non-school based with community partners, and non-school based without community partners.

This research builds upon previous analyses of the YEAH! Program discussed in Botchwey [5] and Millstein, et al [19]. While the previous research focused on student outcomes by race/ethnicity, sex, and geography, this research focuses on student outcomes by club type. The study design, recruitment, retention, and data collection are consistent while the statistical analysis is unique. More information about the design of the YEAH! program is included in earlier papers [5,18]. Students presented a built environment advocacy project to a decision-maker, either the principal of the school, the parks and recreation director for the city or another project-related leader.

### 2.1. Recruitment

The study team recruited youth serving organizations located in low-income urban, suburban and rural communities with a large representation of the target race/ethnicity groups. Organizations received mailed and emailed promotional materials, phone calls and vists from the study team. Very few organizations joined as a result of this approach, instead, the study team revised it’s recruitment protocol based on places and institutions where we could build on past relationships. The study team focused on organizations located in the communities that met the inclusion criteria, and those that we were specifically referred to by national and local youth-serving organizations, community leaders, and both the study team and YEAH! advisory board’s networks. Each organization identified and adult leader to serve as the study team’s point of contact and the YEAH! club leader who was responsible for recruiting youth, collecting forms and surveys and delivering the curriculum. The study team collected consent forms from adult leaders and provided training on the curriculum and study. The adult leaders collected all consent forms from youth and guardians, directing questions to the study team. Consent forms were available in English, Spanish and Chinese. All procedures followed the study’s approved IRB (Protocol H16465) [18].

### 2.2. Participants

The sample used in this analysis included *n* = 116 middle school-aged (range: 11–14 years old; mean = 12.20; SD = 0.94 minority youth within 12 youth-serving organizations (e.g., schools, Boys and Girls Clubs, Big Brothers Big Sisters) throughout the U.S. This is a subset of the *n* = 137 students and 18 organizations examined in Botchwey et al [5]. The previous analysis examined student outcomes clustered by race/ethnicity, sex, and geography with a minimum of sixteen students in each category. This analysis examines student outcomes clustered by club. To ensure that the results were reliable, clubs with less than six students were dropped from the analysis. Six clubs and a total of twenty-one students included in the previous analyses were not included in this analysis.

The students completed pre- and post-intervention surveys allowing for matched-pair comparisons for changes in physical activity and behaviors over time. Different from the Botchwey et al, study [5], which examined students’ pre-post intervention changes in YEAH! outcomes by race/ethnic groups and place/geography (urban, suburban, rural areas), the analyses for this paper focused on students’ pre-post intervention changes in YEAH! outcomes grouped by the characteristics of the club conducting the advocacy intervention.

Four classifications for club types were defined: (1) School-based club with a partner, (2) Non-school-based club with a partner, (3) Non-school-based club without a partner, and (4) school-based clubs without a partner. School-based clubs are those that participated in the ten-week program within a school setting, either during the school day or after school for students at the middle school level (5th–8th grade depending on the district). Non-school based clubs are other youth-serving organizations that take place off school sites including Boys and Girls Clubs, churches, and YMCAs. Program facilitators and researchers based at Georgia Institute of Technology who interacted regularly with adult leaders for recruitment, retention, and overall support coded clubs as having or not having external partnerships. Schools or non-school based clubs that were a part of a network of organizations advocating for healthier youth were defined as clubs with a partnership. Examples of these partnerships include city or county health department programming. As shown in Table A1 (see Appendix A), there were 3 school-based clubs with partners (25 students), 3 non-school-based clubs with a partner (23 students), 2 non-school-based clubs without a partner (24 students), and 4 school-based clubs (44) students.

### 2.3. Data Collection and Measures

Students completed pre- (1st week) and post-intervention (10th week) assessments that were based on validated YEAH! questionnaire measures [19]. There were 13 subscales measured twice (pre- and post-intervention repeated measures) and 10 post-only subscales assessed only once (post-intervention). All survey questions addressed areas hypothesized to be influenced by participation in the YEAH! program [19].

As shown in Table A2, the questions in the survey were grouped based on the subscales items fell on per the confirmatory factor analyses (CFA) and inter-item correlations reported in Millstein et al [19]. All survey questions (except for those in the “Nutrition and Physical Activity” section in Table A2) had survey response options on a Likert scale that ranged from 1 (strongly disagree) to 5 (strongly agree). For subscales in which questions were asked in a negative direction relative to the scale’s interpretation, such as the ‘active participation’ subscale, item responses were reverse-coded so that higher values indicated more agreement and lower values more disagreement with the scale construct. Participants’ subscale scores were computed as the mean of the item responses to the questions in each subscale.

The exceptions to the ‘disagree-agree’ response options were for the questions in the “Nutrition and Physical Activity” section. Item responses to these questions were used to create subscale scores that reflected days per week, servings, or counts as appropriate for the questions (see Table A2). The paper-based surveys took approximately twenty minutes to complete. More detailed information about the survey instrument design and subscales can be found in Botchwey et al [5].

### 2.4. Statistical Analyses

All data analyses were conducted in IBM SPSS Statistics for Windows, version 25 (IBM Corp., Armonk, N.Y., USA). Descriptive statistics examined the distribution of all measures to ensure there were no improbable outliers. The SPSS Mixed linear regression procedure was used to examine all YEAH! subscales as dependent measures, so that any covariation due to clustering of participants recruited within clubs/organizations could be adjusted for as a random effect in the models. For the 13 subscales measured twice, the mixed models included both inter- and intra-group effects to assess mean differences (i.e., between-groups effects) across the 4 club types (school-based with a partner, non-school based with a partner, and non-school-based without a partner, and school-based without a partner), mean changes from pre- to post-intervention (repeated measures effects), and mean differences in average pre-post changes over time by club-type (i.e., group-by-time interaction effects). For the 10 subscales that were measured only once post-intervention, mixed regression models assessed mean differences across club type (i.e., between-groups effects only). Given this paper’s primary aim of assessing potential differential impact of youth advocacy training conducted by different types of clubs, the club type-by-time interaction effects were the key results for the 13 subscales measured both pre- and post-intervention, and the club type between-groups effects were the key results for the 10 subscales measured post-intervention only. Per the exploratory nature of this paper, key effects that showed significant results (*p* < 0.05) or “trends” (*p* < 0.20) were described and patterns illustrated in the figures.

## 3. Results

Table A3 shows the mixed-model results for each of the 23 YEAH! subscales analyzed. The statistical significance of the club-type main effects (i.e., between-groups club characteristics categories) are shown for all subscales, including the 13 subscales measured twice and the 10 subscales measured only once post-intervention. For the 13 subscales measured twice, this table also provides results for the pre-post changes (i.e., within-person time effects), the interactions of time-by-club type, and the direction of change for the estimated marginal means for pre- and post-intervention variables. 

Per the primary aim of examining differential impact on advocacy training outcomes depending on the type of club/organization where the training occurred, the key model effect of interest for the 13 outcomes measured twice was the club type-by-time interaction effect. For the 10 outcomes measured only once, the between-groups main effect of club type was the key result indicative of average differences across club types on the outcome measures only relevant post-intervention. The model effects that were significant (*p* < 0.05) or represented “trends” (*p* < 0.20) were graphed to illustrate patterns by club types.

**Figure 1:**
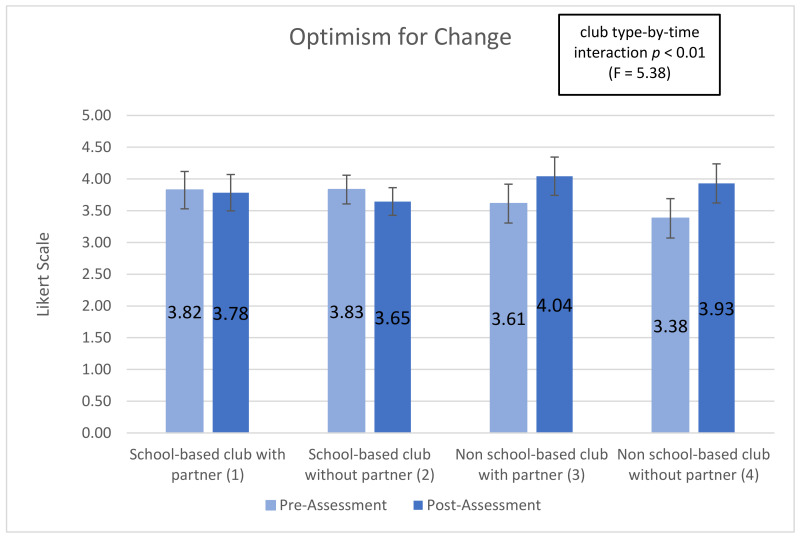
Optimism for Change pre- post-assessments by club type and partnership.

**Figure 2:**
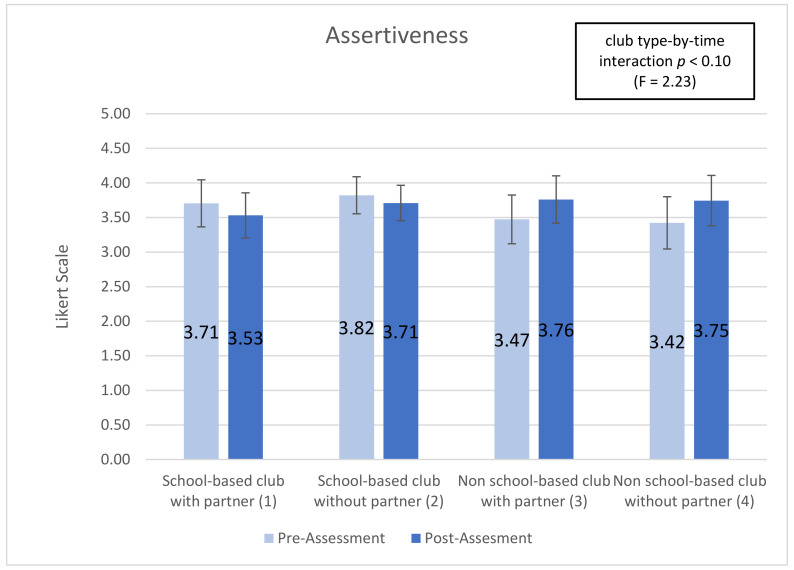
Assertiveness pre- post-assessments by club type and partnership.

**Figure 3:**
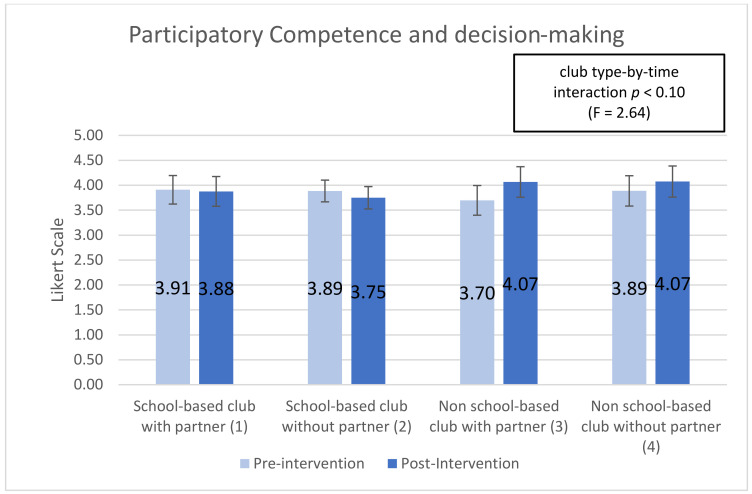
Participatory Competence and decision-making pre- post-assessments by club type and partnership.

**Figure 4:**
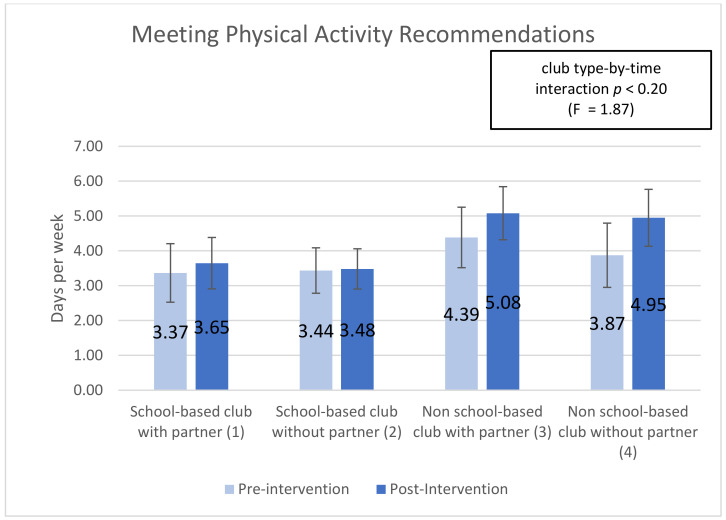
Meeting Physical Activity Recommendations pre- post-assessments by club type and partnership.

**Figure 5:**
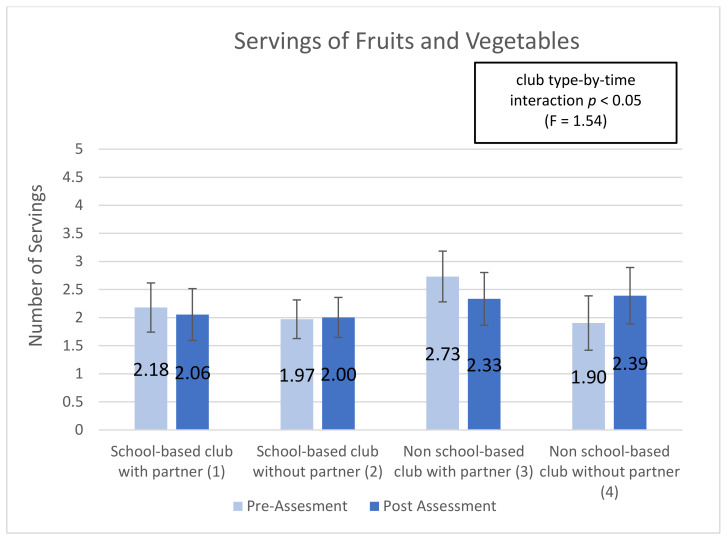
Servings of Fruits and Vegetables pre- post-assessments by club type and partnership.

**Figure 6:**
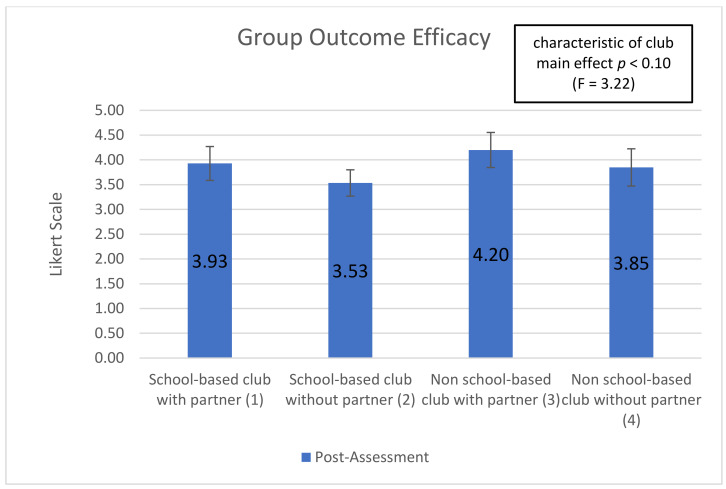
Group Outcome Efficacy post-assessment by club type and partnership.

**Figure 7:**
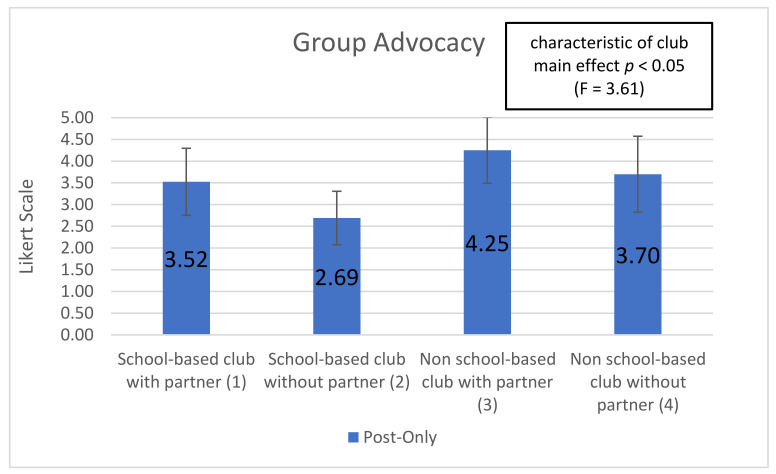
Group Advocacy post-assessment by club type and partnership.

Five subscales showed significant (or trend) time-by-club type interactions, indicating that club types were associated with differential changes in the subscale over time (interaction effect): ‘optimism for change’ (*p* < 0.01), ‘assertiveness’ (*p* < 0.10), ‘participatory competence and decision-making’ (*p* < 0.10), ‘meeting physical activity recommendations (*p* < 0.20), and ‘servings of fruits and vegetables’ (*p* < 0.05). Additionally, 2 of the 10 post-intervention-only subscales showed significant main effects of club type: ‘group outcome efficacy’ (*p* < 0.10) and ‘group advocacy’ (*p* < 0.05). Mean patterns across club types are shown in Figure 1, Figure 2, Figure 3, Figure 4, Figure 5, Figure 6 and Figure 7.

The ‘optimism for change’ subscale is a Likert scale variable that measures youth perception that leaders in their school or community listen to them and the belief that youth have a say in their community. There is a statistically significant (*p* < 0.01) time-by-club characteristic interaction for the ‘optimism for change’ subscale. Students in school-based clubs with partner declined from 3.824 to 3.784 (−1.04%); students in school-based clubs without a partner declined from 3.833 to 3.645 (−4.9%); students in non-school-based clubs with partner increased from 3.612 to 4.043 (+11.93%); students in non-school based clubs without partner increased from 3.380 to 3.930 (+16.27%) (see Figure 1). The mean change from pre-to-post assessment for students in non-school based clubs compared to school-based clubs had a different direction and different magnitude.

The ‘assertiveness’ subscale is a Likert scale variable that assesses students’ confidence in their ability to communicate with adults about issues, to ask others for help working toward a healthier community, and to initiate conversations about built environment changes. The ‘assertiveness’ subscale has a trend (*p* < 0.10) significance for the interaction of time-by-club characteristics. Students in school-based clubs with partner declined from 3.705 to 3.531 (−4.69%); students in school-based clubs without partner declined from 3.822 to 3.710 (−2.9%); students in non-school-based clubs with partner increased from 3.473 to 3.761 (+8.29%); students in non-school-based clubs without partner increased from 3.38 to 3.93 (+9.26%) (see Figure 2). This research includes trend values (*p* < 0.10) because it is exploratory. 

The “participatory competence and decision making” variable measures students” perception of their ability to persevere beyond challenge and influence decisions in group settings. On average students across club types felt fairly confident in their ability to overcome challenges. However, students in school-based clubs declined 0.81% and 3.5% for clubs with a partner and without a partner, respectively. While students in non-school based clubs increased 9.9% in non-school based clubs with partners and 4.8% in non-school based clubs without partners (see Figure 3). The differences in mean pre-to-post assessment scores for students in different club types (school-based with and without a partner and non-school based with and without a partner) showed a trend (*p* < 0.10) significance. The direction and magnitude of change between the four club types across time was significant enough at the trend level to be included in the paper.

The ‘meeting physical activity recommendations’ subscale measures student’s self-reporting of the number of days (seven weekdays) for which they were active for at least sixty minutes per day pre- and post-YEAH! Intervention. On average, students in school-based clubs were physically active two days less than students in non-school based clubs. As found in the prior YEAH! Study [5] the YEAH! Curriculum increases the number of days students are physically active for at least 60 min per day. This research builds on that finding, specifying that students in non-school based clubs with and without partnerships had a larger increase in the number of physically active days after participating in YEAH! Students in school-based clubs with a partner increased 8.3% (3.36 to 3.64), this was the largest increase among students in school-based clubs as students in school-based clubs without a partner increased just 1.3% remaining at an average of 3.4 days. Students in non-school based clubs with a partner increased 15% from 4.38 to 5.0 days on average. While students in non-school based clubs without a partner increased 27% from 3.8 to 4.9 days on average (see Figure 4). The ‘meeting physical activity recommendations’ subscale was the least statistically relevant across time-by-club characteristic interaction (*p* < 0.20), but it was statistically significant (*p* < 0.05) for time and club characteristic. This demonstrates that difference in the mean from pre-to-post assessment by club characteristic (time-by-club characteristic interaction) was weak. However, from pre- to post- assessment (time) student mean value changed and that students in different club types had different changes (club characteristic).

“Servings of fruits and vegetables” measures a typical day’s consumption of fruits and vegetables self-reported by students on pre- and post-assessment forms. The “servings of fruits and vegetables” subscale is statically significant (*p* < 0.05) for time-by-club characteristic interaction. Daily servings of fruits and vegetables were generally low among all students, averaging 2 servings per day. Students in school-based clubs with partner declined from 2.180 to 2.055 (−5.7%); students in school-based clubs without partner increased from 1.972 to 2.004 (+1.62%); students in non-school-based clubs with partner declined from 2.733 to 2.334 (−14.59%); students in non-school-based clubs without partner increased from 1.904 to 2.391 (+25.57%). Although significant, student access to partnerships appears to have no positive impact on their increased or decreased servings of fruits and vegetables (see Figure 5).

Two of ten post-only subscales were statistically relevant for this exploratory paper. For both subscales, the club characteristic main effect was used as the standard for measurement. These variables are Likert scale measures that include questions that were presented to students only after program completion. They assess not only student’s opinions about the YEAH! curriculum but also the efforts of their club. The ‘group outcome efficacy’ subscale (*p* < 0.10) measures students’ opinions about the ability of their group to influence how adults and their peers feel about nutrition and physical activity. Non-school-based clubs with a partner had the highest positive perception of their group’s ability to influence opinions than the other clubs at 4.20, school-based clubs without a partner had the lowest positive perception at 3.534. Students in school-based clubs with a partner were slightly more positive (3.928) than students in non-school-based clubs without partners (3.848) (see Figure 6).

The final post-only variable ‘group advocacy’ (*p* < 0.05) measures students’ feelings about the way that their final presentation and advocacy were received by decision-makers. The ‘group advocacy variable asked students to rate their agreement on a Likert scale of 1 (strongly disagree) to 5 (strongly agree). Students were asked if the decision-maker listed carefully, seemed to understand the requests, seemed to learn something from the presentation, was impressed, and if the decision-maker was going to make changes based on the presentation. Students’ answers to these questions that constitute the ‘group advocacy’ subscale demonstrate their perceived influence on adult decision-makers and the extent to which their efforts were received. Students in non-school-based clubs with a partner had the highest positive perception of their experience presenting to a decider (4.252) compared to students in school-based clubs without a partner which had the most negative perception of the experience (2.690) (see Figure 7).

## 4. Limitations

This study recruited extensively from neighborhoods and youth serving organizations with a focus on low-income minority communities. There were some recruitment, retention and programmatic challenges that were also documented in the first YEAH! evaluation [20] due to changes in leadership in the partner organizations. We had two instances where adult leaders left the non-profit partner organization, and so these two locations did not complete participation in the study and were not included in the final analysis.

- Another possible limitation is the self-reported nature of the survey data. Youth are asked to self-report their experiences in the pre and post surveys, however we feel the youth are best qualified to report on their feelings of well-being, leadership, and their perceived changes in themselves and their communities.
- While this study showed strong recruitment from diverse populations, including African American, Latinx, and Asian American groups, the recruitment of Pacific Islander and Native/Indigenous youth for participation was more challenging. While the study authors are aware of distinct outcomes around health and activity between and among different Asian and Pacific Islanders [21] the two groups were aggregated for analysis due to a high self-reporting of “multi-racial” identity among a majority of youth participants in Hawaii (a primary recruitment site for this study), including mixed-race identities of white, Native-Hawaiian, Pacific Islander, and Asian [22].
- Analyses for this paper were considered exploratory and *p*-values for significance tests relaxed; therefore, highlighting results that only showed weak “trend” effects was a limitation. Such trends should be considered suggestive of underlying effects, and they call for replication in other research to confirm.

## 5. Discussion

The aim of this paper is to assess the impact of two general characteristics of clubs: the type of club (whether that club is school-based or non-school based) and partnership (presence or absence a community partnership) on student advocacy and health outcomes after participating in the 10-week YEAH! curriculum. The research finds that students non-school based clubs regardless of the presence of a community partner had better outcomes in terms of youth psychosocial outcomes. Furthermore, we find that the presence of community partner in school-based clubs is critical for students to meet the advocacy goals of YEAH!

Research has examined the benefits and challenges community-based and school-based after-school programs together, often comparing ASP to non-ASP students. It is worthwhile to consider differences in program design and program reach between community-based and school-based after-school programs. School-based programs focus more on academic activities, engage with the student’s teacher, and serve academically challenged youth. Community-based programs engage in more social activities, engage with the student’s parent, and serve youth with behavioral challenges. School-based programs are shorter-term, typically lasting for a semester while community-based programs last over multiple years. The differences in program design result in school-based after-school programs having greater positive outcomes on student academic performance (grades, attendance, attitude toward education) and community-based programs having greater positive impacts on student youth and social development (self-esteem, communication, concern for others) [23]. Research has shown that after-school programs differ widely in terms of program design and program outcomes.

Youth psychosocial outcomes measure the interaction of social elements, thought, and behaviors. In the YEAH! curriculum these are measured by 11 subscales shown in Table A2. Three subscales were statistically significant in this research, “optimism for change”, “assertiveness”, and “participatory competence and decision-making”. Students in non-school based clubs had positive increase in all three variables while students in school-based clubs declined (results are shown above). As measured by “optimism for change” non-school based club students increased between 11% and 16% while school-based club students decreased between 1% and 4%. In the “assertiveness” variable non-school based students increased around 9% while school-based students decreased between 2% and 4%. Finally, as measured by “participatory competence and decision-making” non-school based students increased between 4% and 8% while school-based students declined between 1% and 3%.

The difference in direction of change (increase or decrease) and magnitude of change between school-based and non-school based students regardless of the presence of a community partnership demonstrate that non-school based clubs, those happening at youth centers or religious organizations help students improve the social and personal growth components of YEAH! better than school-based clubs. The elements of YEAH! which focus on optimism for a better future, confidence, perceived control over ones outcome, and perseverance are improved among non-school based students while these social skills decline among students in school-based settings (see Appendix A). After school programs generally can help with personal and social skill development among adolescent aged youth, but questions among researchers remain around why some ASPs are more effective than others [12,24].

The post-only subscales measure student perception after participating in YEAH! Two of the ten post-only measures were statistically relevant in this research, “group outcome efficacy” and “group advocacy”. Students in school-based clubs without a partner fared the worst in these advocacy and advocacy outcome metrics. YEAH! is designed so that students select a community advocacy project (park, school, store, street, etc.) and present their recommendation for approval to a “decision-maker” such as a principal, public administrator, or elected official. YEAH! club projects focused on park improvements, adding dance classes to school curriculum and access to clean water fountains to rehydrate after play. These two variables ask students to measure the extent to which their “decision-maker” listened to them, understood their recommendation, was impressed by their recommendation, and make changes based on their recommendation (group advocacy). Additionally, the “group outcome efficacy” subscale asks students to assess their perceived influence over their peers and adults beliefs about physical activity and nutrition. Students in non-school based clubs with and without a partner as well as students in school-based clubs with a partner had significantly better perceptions of advocacy and efficacy than students in school-based clubs without a partner. This research demonstrates that school-based clubs must have a community partner when implementing YEAH! in order to have positive student perception of the effectiveness of their advocacy. This makes sense for practical reasons, if the club is focusing on tobacco advertisements in a neighborhood store it is difficult for students to conceptualize the impact that their principal, the ‘decision-maker’, will have on making a change.

In addition to school-based and community-based after-school programs, there are three identified program models: mentoring, rites of passage, and extracurricular activities [8]. The mentoring model focuses on building close connections between youth and supportive adults. Benefits associated with the mentoring model of after-school programs are reduced absenteeism, reduced violent behavior, and decreased first-time drug usage compared to students not participating in the program [25].

The rites of passage model is particularly relevant to ethnic minorities such as African Americans. Programs following this model seek to develop students’ knowledge of their collective history and culture and help them become productive members of society. The rites of passage model is shown to minimize risky behavior among Black/African American males and increase cultural pride, agency, and self-esteem [26,27]. The extracurricular model is the most well known, it focuses on skill-based programming such as sports, arts, science, mathematics. After-school programs with an extracurricular model typically are closely tied to schools.

The YEAH! curriculum measures health outcomes through self-reported physical activity and nutrition. The impact of community partnerships and club type had little impact on student physical activity after participating in YEAH! Prior iterations of this research [18] found that YEAH! participants on average (regardless of race/ethnicity, sex, or neighborhood type) increased the number of days physically active for at least sixty minutes per day.

Whereas non-school based afterschool programs bring together in students of color and low-income students from the larger metropolitan area or different rural/urban/suburban schools, participation in YEAH! provides an opportunity outside of school to work on a project together and share an experience. This may be one factor that is connected to the significant positive outcomes in students increased optimism, confidence, and assertiveness in non-school based programs.

## 6. Conclusions

In this paper, we examine the role of peer and group influence and the relationship to individual feelings of empowerment and advocacy in their community. We explore the relationship between club type (school-based and non-school based) and community partnership (presence and absence). We find that when YEAH! students participate non-school based clubs, with or without a community partnership, they have better social and emotional outcomes such as confidence, empowerment, and perseverance to achieve a goal. Additionally, we find that community partnerships are essential to students receiving the full benefits of the advocacy elements of YEAH!

Missing from the public debate and academic scholarship concerning challenges of instrument effectiveness is a robust discussion of how focusing on self-advocacy, youth participation and youth empowerment can improve collective health outcomes. These two approaches could be considered as principal mechanisms for measuring and promoting physical activity behavior change, as well as change within the physical activity environment.

The authors show evidence of a clear need to unite analyses of place/geography (suburban, rural, urban) traits with other mechanisms of behavior change in future attempts to promote youth physical activity. These mechanisms include social and psychological variables like self-efficacy, self-image, and self-confidence. These qualitative, behavior targeted efforts to improve youth physical activity had direct relationships with improvements in not just overall physical activity—but also hopefulness for seeing change in the built environment.

Our study demonstrates how important after-school activity sites have been as an equalizer for children with limited access to parks or walkable neighborhoods, and with the almost uniformly nationwide closure of these resources, coupled with existing inequality of access, we use our existing data to provide an analysis of how the closure of public-facing resources (natural amenities and social ones such as schools and afterschool recreation sites) could disproportionately impact urban students over rural or suburban ones. In a previous study [5] we found that urban students had the lowest levels of “hope” or what we termed “optimism for change”. What types of partnerships will be required to continue to improve outcomes around hope, and maintain equitable access to physical activity spaces, through current and future times of pandemic and civil unrest?

It is clear that afterschool programs overall have significant benefits for students [2,12]. However, it is also important to examine how ongoing disparities in physical access and social environments determine overall neighborhood and community health. Where City Planning and Public Health can and do intersect is the incorporation of policy-oriented thinking ahead; we may operate within the framework of existing inequalities but with the optimism and belief that we can change adverse outcomes. As planners in the public health field, we are also asking about participation: what role do youth have in creating more self-determined spaces, beyond existing inequitable access? We join others in asking what role youth, specifically youth of color, may have in the design of our future communities [28]. To that end, planners or other third-party agencies and non-governmental organizations, partnering with after school programs that have access to youth—but not necessarily access to spaces of urban planning—is an important connection and intervention that is unique to the YEAH! curriculum.

Future research on this topic should focus on how youth can participate in the process of city planning (thus becoming their own advocates) for healthier communities. Our findings show improvements in individual health (in terms of nutrition) can be connected to positive feelings about authority, choice, and power in decision-making. We believe this is especially important for youth of color—not only for their current outcomes but for future outcomes and future participation in planning for healthier communities.

## Data Availability

Data supporting reported results can be found by contacting the study authors.

## Appendix A

**Table A1:** Distribution of students across club types, with “x” indicating which type the club represented.

Club Number	Count of Students	School-Based Club with a Partner	School-Based Club without a Partner	Non-School-Based Club with a Partner	Non-School-Based Club without a Partner
1	6	x			
2	9	x			
3	17				x
4	7			x	
5	7			x	
6	9			x	
7	7				x
8	10	x			
9	11		x		
10	7		x		
11	14		x		
12	12		x		
Total:	116	25	44	23	24

**Table A2:** Survey questions for each subscale.

Theme	Subtheme	Subscales (Number of Representative Questions)	Questions Used for Each Subscale	Pre-Post Measures	Post Only Measures
Intervention Processes (IP)	Intervention Processes (IP)	Group cohesion (2)	“1. Members of our group do not spend time together outside of meetings or events. 2. I’m unhappy with my group’s level of commitment to its goals for creating healthier communities”		X
		Roles and participation (2)	1. When I attended meetings, I took part in the discussions. 2. I took responsibility for things that the group needs to have done		X
		Opportunities for control in group work (2)	“1. This group allowed me to have a say in planning events or activities. 2. This group had specific leadership roles for youth”		X
		Coordinator/leader characteristics (3)	“1. Our leader(s) provided help whenever we needed it. 2. Our leader(s) did not force his or her ideas and opinions on the group 3. Our leader(s) let us work through our disagreements to decide what was best for the group ”		X
		Group resiliency (2)	1. This group does not give up during tough times. 2. If this group failed to accomplish one of our goals, we kept trying to find a way to reach it.		X
Youth Psychosocial Factors, Participation, and Hypothesized Drivers of Change (YPF)	Perceptions	Self-efficacy for health and advocacy behaviors (3)	1. I am sure that I can tell my friends to eat healthily. 2. I am sure that I can tell my friends to be physically active. 3. I am confident that I can work to make my school or community a better place for being physically active and eating healthy.	X	
		Active participation (2)	“1. I like to wait and see if someone else is going to solve a problem. 2. I find it very hard to talk in front of a group.”	X	
		Optimism for change (2)	“1. If I tell someone “in charge”, like a leader, about my opinions, they will listen to me. 2. I enjoy participation because I want to have as much say as possible in my school or community.”	X	
		Peer support for healthy behaviors (2)	1. How many of your five closest friends are physically active at least 5 days a week? 2. How many of your five closest friends eat at least 5 servings of fruits and vegetables a day?	X	
		Advocacy outcome efficacy (1)	1. This project can make a difference in making our school or community a better place for being physically active and eating healthy.	X	
		Group resiliency	1. This group does not give up during tough times. 2. If this group failed to accomplish one of our goals, we kept trying to find a way to reach it.		X
	Knowledge and Skills	Assertiveness (3)	“1. I can talk with adults about issues I believe in. 2. I can ask others to help work on making our school or community healthier. 3. I can start discussions with others about how to change our school or community to make it healthier.”	X	
		Participatory competence and decision-making (2)	1. If I have a problem when working towards a goal, I usually do not give up. 2. I can influence the decisions my group makes.	X	
		Pride in group work (2)	1. I am proud of the work our group did. 2. Our work was worth the time and effort we put into it.		X
		Group outcome efficacy (2)	“1. This group can influence how adults in the community feel about nutrition and physical activity. 2. This group can influence how people my age, who are not in this group, feel about nutrition and physical activity.”		X
		Health advocacy history (2)	“1. In the last year, how many times have you tried to tell other students, your family, or friends to think more about eating healthy or being physically active. 2. In the last year, how many times have you tried to tell school leaders, people in your community, or politicians to be more interested in making your school or community a better place for being physically active and eating healthy.”	X	
Proximal Outcomes: Individual Youth Changes (PO)	Nutrition and Physical Activity	Meeting physical activity recommendations (2)	1. Over the past seven days, how many days were you physically active for at least 60 min per day? 2. Over a typical week, on how many days are you physically active for at least 60 min per day?	X	
		Sports/Enjoyment of physical activity (2)	“1. Not counting PE classes, how many days per week do you play or practice a team sport, or take a physical activity class? 2. I enjoy physical activity.”	X	
		Active transportation (2)	1. In a typical week, how many days do you walk or bike TO school? 2. In a typical week, how many days do you walk or bike FROM school?	X	
		Servings of fruits and vegetables (2)	1. In a typical day, how many servings of fruit do you eat? 2. In a typical day, how many servings of vegetables do you eat?	X	
		Fast food servings/week	1. Outside of school, how many times per week do you eat fast-food?	X	
	Advocacy Related	Intent to remain involved (2)	“1. I plan to continue to work for change in my school or community after this project is over. 2. If I had a chance to join a similar group in the future, I would do it.”		X
		Group advocacy (6)	“1. The decision-maker(s) listened carefully to our group. 2. The decision-maker(s) seemed to understand what we were asking for. 3. The decision-maker(s) seemed to learn something new from what we were saying. 4. The decision-maker(s) would have listened to us more if we were adults instead of youth. 5. The decision-maker(s) were impressed by our group’s work. 6. The decision-maker(s) are going to make some changes based on the information from our group.”		X
		Personal advocacy activities since starting YEAH! (2)	“1. Since I started this project, I have talked to my parents or family members about changes needed to make my school or community a better place for being physically active and eating healthy. 2. Since I started this project, I have talked to my friends about changes needed to make my school or community a better place for being physically active and eating healthy.”		X

**Table A3:** Pre/Post-Intervention and Post-Intervention-only YEAH! Subscales: Effects of Club Type, Time, and Time-by-Club Type Interactions.

		Estimated Marginal Means (Standard Error)				Main Effects		Interaction
Subscale	Time	School-Based Club with a Partner	School-Based Club without a Partner	Non-School-Based Club with a Partner	Non-School-Based Club without a Partner	Time (Pre-Post)	Characteristics of Club	Time * Characteristics of Club
Pre-post subscales								
Youth psychosocial factors, participation, and hypothesized drivers of change (YPF) perceptions								
Self-efficacy for health and advocacy behaviors	Pre-intervention	3.783(0.174)	3.717(0.137)	3.900(0.178)	4.017(0.194)	-	-	-
	Post-intervention	3.970(0.184)	3.581(0.145)	4.007(0.192)	4.104(0.206)			
	Direction of change	I	D	I	I			
Active participation	Pre-intervention (1)	3.492(0.188)	3.445(0.140)	3.130(0.192)	3.163(0.192)	8.403 **	-	-
	Post-intervention (2)	2.825(0.202)	2.704(0.152)	2.978(0.207)	2.946(0.210)			
	Direction of change	D	D	D	D			
Optimism for change	Pre-intervention	3.824(0.147)	3.833(0.113)	3.612(0.153)	3.380(0.155)	5.414 *	-	5.384 **
	Post-intervention	3.784(0.143)	3.645(0.109)	4.043(0.151)	3.930(0.154)			
	Direction of change	D	D	I	I			
Peer support for healthy behaviors	Pre-intervention	2.656(0.239)	2.512(0.184)	3.000(0.248)	2.830(0.252)	10.235 **	3.358 **	-
	Post-intervention	2.939(0.220)	2.714(0.167)	3.783(0.229)	3.106(0.235)			
	Direction of change	I	I	D	D			
Advocacy outcome efficacy	Pre-intervention	4.55(0.153)	4.348(0.117)	4.087(0.159)	4.379(0.163)	3.640 +	-	-
	Post-intervention	4.159(0.170)	4.021(0.130)	4.104(0.181)	4.274(0.183)			
	Direction of change	D	D	I	D			
Assertiveness	Pre-intervention	3.705(0.170)	3.822(0.134)	3.473(0.176)	3.423(0.189)	-	-	2.229 +
	Post-intervention	3.531(0.163)	3.710(0.128)	3.761(0.171)	3.745(0.182)			
	Direction of change	D	D	I	I			
Participatory competence and decision-making	Pre-intervention	3.909(0.143)	3.885(0.109)	3.697(0.149)	3.887(0.152)	1.779 ++	-	2.635 +
	Post-intervention	3.877(0.149)	3.749(0.112)	4.066(0.153)	4.074(0.156)			
	Direction of change	D	D	I	I			
Health advocacy history	Pre-intervention	1.494(0.261)	1.693(0.211)	2.085(0.267)	1.634(0.298)	-	-	-
	Post-intervention	1.674(0.243)	1.860(0.200)	1.998(0.247)	1.759(0.281)			
	Direction of change	I	I	D	I			
Proximal outcomes: individual youth changes (PO) nutrition and physical activity								
Meeting physical activity recommendations	Pre-intervention	3.366(0.420)	3.435(0.326)	4.385(0.434)	3.873(0.461)	9.009 **	3.492 *	1.873 ++
	Post-intervention	3.646(0.369)	3.481(0.289)	5.080(0.381)	4.949(0.408)			
	Direction of change	D	I	I	I			
Sports/Enjoyment of physical activity	Pre-intervention	2.752(0.311)	3.309(0.253)	3.273(0.317)	3.194(0.357)	-	-	-
	Post-intervention	2.793(0.306)	3.300(0.248)	3.469(0.312)	3.444(0.351)			
	Direction of change	I	D	I	I			
Active transportation	Pre-intervention	0.916(0.404)	0.572(0.332)	1.236(0.410)	1.254(0.472)	-	-	-
	Post-intervention	0.716(0.425)	0.697(0.347)	1.019(0.432)	1.317(0.491)			
	Direction of change	D	I	D	I			
Servings of fruits and vegetables	Pre-intervention	2.180(0.219)	1.972(0.172)	2.733(0.226)	1.904(0.242)	-	-	1.545 *
	Post-intervention	2.055(0.231)	2.004(0.178)	2.334(0.235)	2.391(0.251)			
	Direction of change	D	I	I	I			
Fast Food Servings/week	Pre-intervention	2.251(0.888)	2.824(0.663)	2.052(0.906)	2.375(0.927)	-	-	-
	Post-intervention	1.770(0.635)	3.226(0.499)	1.661(0.660)	2.564(0.689)			
	Direction of change	D	I	D	I			
Post-intervention only subscales								
Intervention Process (IP)								
Group cohesion	Post-Only	4.124(0.255)	3.556(0.209)	3.680(0.263)	3.902(0.298)	N/A	-	N/A
Roles and participation	Post-Only	2.193(0.178)	2.261(0.132)	2.344(0.181)	2.242(0.185)	N/A	-	N/A
Opportunities for control in group work	Post-Only	3.910(0.157)	3.705(0.121)	3.861(0.167)	3.886(0.170)	N/A	-	N/A
Coordinator/leader characteristics	Post-Only	4.533(0.153)	3.825(0.117)	4.438(0.159)	4.144(0.167)	N/A	-	N/A
Group resiliency	Post-Only	4.280(0.168)	3.491(0.129)	3.827(0.179)	3.922(0.185)	N/A	-	N/A
Youth psychosocial factors, participation, and hypothesized drivers of change (YPF) perceptions								
Pride in group work	Post-Only	4.663(0.155)	3.898(0.118)	4.429(0.162)	4.306(0.167)	N/A	-	N/A
Group outcome efficacy	Post-Only	3.928(0.171)	3.534(0.133)	4.200(0.177)	3.848(0.188)	N/A	3.215 +	N/A
Proximal outcomes: individual youth changes (PO) advocacy related								
Intent to remain involved	Post-Only	3.744(0.174)	3.690(0.140)	3.865(0.181)	3.849(0.203)	N/A	-	N/A
Group advocacy	Post-Only	3.524(0.386)	2.690(0.308)	4.252(0.381)	3.699(0.437)	N/A	3.611 *	N/A
Personal advocacy activities since starting YEAH!	Post-Only	3.148(0.215)	3.170(0.169)	3.569(0.226)	2.862(0.241)	N/A	-	N/A

*p* < 0.20 ++; + *p* < 0.10; * *p*< 0.05; ** *p* < 0.01. Direction of Change: I = Increase D = Decrease.
